# *Salmonella* contamination, serovars and antimicrobial resistance profiles of cattle slaughtered in South Africa

**DOI:** 10.4102/ojvr.v83i1.1109

**Published:** 2016-05-26

**Authors:** Evelyn Madoroba, Daniel Kapeta, Awoke K. Gelaw

**Affiliations:** 1 Bacteriology Section, Agricultural Research Council-Onderstepoort Veterinary Institute, South Africa; 2 College of Agriculture and Environmental Sciences, University of South Africa, Florida Campus, South Africa

## Abstract

Antimicrobial resistant *Salmonella* are among the leading causes of foodborne infections. Our aim was to determine *Salmonella* contamination during cattle slaughter in South African rural abattoirs (*n* = 23) and environmental samples. Furthermore, antimicrobial resistance patterns of the *Salmonella* isolates were determined. Samples of cattle faeces (*n* = 400), carcass sponges (*n* = 100), intestinal contents (*n* = 62), hides (*n* = 67), and water from the abattoirs (*n* = 75) were investigated for *Salmonella* species using microbiological techniques and species-specific polymerase chain reaction targeting the *invA* gene. In total 92 *Salmonella* species isolates were recovered. The *Salmonella* mean frequency of occurrence on hides, carcasses, and intestinal contents was 35.37% (*n* = 81). Eleven faecal samples (2.75%) tested positive for *Salmonella.* The predominant serovar was *Salmonella* Enteritidis. Diverse serovars that were identified on carcasses were not necessarily found on the hides and intestinal contents. The inconsistent occurrence of the diverse *Salmonella* serovars on hides, carcasses, and intestinal contents implies that in addition to carriage on hides and in intestinal contents, other external factors also play an important role regarding carcass contamination. The 92 *Salmonella* were serotyped and tested for susceptibility towards the following antimicrobials: ampicillin, cefotaxime, enrofloxacin, kanamycin, and oxytetracycline using the disk diffusion method. Most *Salmonella* (*n* = 66; 71.7%) isolates were resistant to at least one antimicrobial with highest resistance observed towards oxytetracycline (51.90%), which highlights the need for strict hygiene during slaughter and prudent antimicrobial use during animal production. In conclusion, cattle slaughtered in South African rural abattoirs harbour diverse *Salmonella* serovars that are resistant to antimicrobials, which could be a public health risk. The findings should assist policymakers with improving implementation of hygienic slaughter of cattle in rural abattoirs, which is paramount from socioeconomic, public health, and epidemiological standpoints.

## Introduction

Bacteria are among the leading causes of foodborne illnesses (Buzby & Roberts 2009). Foodborne outbreaks result in socioeconomic challenges as a result of hospitalisations and associated medications, particularly among the vulnerable groups such as the elderly and immunocompromised individuals (Gragg *et al*. 2013). Among the bacteria, *Salmonella* has been frequently linked to gastroenteritis worldwide (Skov *et al*. 2007). *Salmonella* causes salmonellosis, which is characterised by nausea, abdominal pain, diarrhoea, and sometimes fever that results in morbidity and in some instances mortality in both animals and human beings (Nørrung & Buncic 2008; Velge, Cloeckaert & Barrow 2005). A study by Majowicz *et al*. (2010) found that, globally, *Salmonella* infection is responsible for an estimated 93.8 million cases of human gastroenteritis and 155 000 fatalities annually.

*Salmonella* has been associated with a number of food-producing animals, which makes animals and their products important sources of human infections (Acha & Szyfres 2001; Davies, Dalziel & Gibbens 2004). The risk of *Salmonella* contamination may be present at any stage of food animal production ranging from the live animal to environmental factors (Alexander, Warnick & Wiedmann 2009; Troutt & Osburn, 1997). At the farm level, cattle hides may become exposed to *Salmonella* through contact with contaminated faeces, feed, or the environment, which poses a risk to food safety if these organisms are transferred on the carcass during slaughter (Arthur *et al*. 2007; Brichta-Harhay *et al*. 2008). Cattle may also be contaminated with *Salmonella* during transportation through contact with faeces of other animals. During slaughter, some procedures such as evisceration and splitting may contribute to carcass contamination (Hui 2012). This is exacerbated by the asymptomatic carrier status of some cattle, which may pose a risk along the food chain (Fegan *et al*. 2005; Tadesse & Tessema 2014). Therefore, some of the sources of *Salmonella* contamination are already present well before the animals are presented for slaughter. In this regard, strict hygienic processes during slaughter procedures are paramount in order to reduce the chances of meat contamination.

In South Africa, the *Meat Safety Act* (No. 40 of 2000) provides for specific requirements on how red meat abattoirs should slaughter cattle. However, in some rural abattoirs meat inspection may not be routinely carried out, which often compromises safe meat handling. This potentially exposes rural communities to various foodborne pathogens including *Salmonella* spp. There is paucity of information regarding prevalence of diseases and hygiene measures taken during cattle slaughter among communal cattle producers. Therefore, it is imperative to obtain such information for better understanding of the potential risk of spreading *Salmonella* from cattle slaughtered in rural abattoirs through the food chain and to institute proper and relevant situation-specific management strategies for foodborne diseases.

The prevalence of antimicrobial resistance among foodborne pathogens has increased during recent decades (Economou & Gousia 2015). The increase in antimicrobial resistance among Gram-negative bacteria such as *Salmonella* may be attributed to the overuse of antimicrobials in food-producing animals for growth promotion, treatment of infection, or for prophylaxis (Economou & Gousia 2015). In South Africa, the *Fertilizers, Farm Feeds, Agricultural Remedies, and Stock Remedies Act* (Act 36 of 1947) makes provision for antimicrobial administration in animals (Henton *et al*. 2011). This allows individuals such as farmers to access stock remedies over the counter (Henton *et al*. 2011), which may contribute to antimicrobial overuse. However, the *Medicines and Related Substances Control Act* (Act 101) also controls veterinary medicines whereby antimicrobials for animal use are only prescribed by a veterinarian (Henton *et al*. 2011).

There is limited information regarding *Salmonella* contamination of carcasses by hides and intestinal contents during cattle slaughter in South African rural abattoirs. Information about antimicrobial resistance of *Salmonella* isolates recovered from cattle in South African rural communities is scant. This study was aimed at contributing to knowledge about the extent of contamination of cattle carcasses by hides and intestinal contents during slaughter and antimicrobial resistance of the *Salmonella* isolates. The objectives of this study were therefore to determine the presence and serovar diversity of *Salmonella* on cattle hides, carcasses, and intestinal contents of cattle slaughtered in rural abattoirs (*n* = 23) of Vhembe district in Limpopo Province of South Africa. In addition, the occurrence of *Salmonella* spp in water samples (used in abattoirs) and fresh cattle faeces from communities that supply rural abattoirs with animals for slaughter was determined in a parallel study. Furthermore, antimicrobial resistance patterns of the *Salmonella* isolates recovered from slaughtered cattle and environmental samples were determined.

## Materials and methods

### Study area and design

Vhembe district municipality is located in the northern part of Limpopo Province of South Africa and it comprises four local municipalities; namely Mutale, Musina, Makhado, and Thulamela. In this study, samples were collected from cattle slaughtered in rural abattoirs of Vhembe district from all the four local municipalities. A cross-sectional study involving 23 rural abattoirs that slaughter cattle was conducted between March 2011 and April 2012. To our knowledge, these represent all the rural abattoirs that had permission to slaughter cattle in Vhembe district during the study period. The names of the abattoirs have been withheld for confidentiality.

Cattle for slaughter at the rural abattoirs were obtained from farms in any of the four local municipalities of Vhembe district. In addition, some of the cattle were brought for slaughter by community members in preparation for events such as weddings, funerals, or other important family functions. The study targeted collection of matched 100 hide samples, 100 carcass swabs, and 100 rectal swabs. The sample size was determined using the following formula described in the European Food Safety Authority (EFSA) Report on ‘Development of harmonised survey methods for foodborne pathogens in foodstuffs in the European Union’ (Käsbohrer *et al*. 2010):

n∞=(Zα)2*p*(1−p)L2 [Eqn 1]

Where *n* = sample size; Z_*α*_ = desired confidence level at 95% (equivalent to Z_*α*_ value of 1.96); and *L* = Accuracy, which was set at 0.05 in this study; *p* = annual expected prevalence.

The annual expected *Salmonella* prevalence in South African cattle is not formally documented, hence an approximate prevalence of 18.75%, which was based on a retrospective study by Kidanemariam, Engelbrecht and Picard (2010) was used in this study. Assuming a prevalence of 18.75%, the estimated sample size is 234, hence this study targeted 300 samples from cattle hides, carcasses, and intestinal contents.

A parallel study involving collection of freshly voided cattle faeces (*n* = 400) was carried out in order to ascertain the possible risk of *Salmonella* shed by cattle in Vhembe district communities and to obtain information about asymptomatic animals that had potential of contaminating carcasses during slaughter. Sample size was also based on the assumptions that were made for slaughtered cattle. Water samples used in the abattoirs (*n* = 75) were also analysed for the presence of *Salmonella*.

### Sample collection

#### Carcasses

Samples were collected from carcasses (*n* = 100) using premoistened commercial beef Carcass Sampling polywipe Kits (ThermoFischer Scientific, Waltham, MA, USA) as described in the United Kingdom Meat Industry Guide. Using a sweeping motion, the sponge was rubbed firmly across the carcass from the hind quarter covering an area of 1000 cm^2^ per carcass. The polywipe sponge was placed in a sample bag, labelled appropriately and kept cool (but not frozen) by immediately placing in insulated cooler boxes containing frozen freezer blocks and transported to the Feed and Food Analysis laboratory, Bacteriology Section of the Agricultural Research Council-Onderstepoort Veterinary Institute (ARC-OVI).

#### Hides

Samples were collected from external hide surfaces (*n* = 67) by rubbing using a premoistened sterile sponge covering 500 cm^2^. Briefly, sterile square metal templates covering 100 cm^2^ areas were placed onto hides and two sponges were used to swab five consecutive areas (Antic *et al*. 2010). Each sponge was placed in a separate stomacher bag and transported to the ARC-OVI Feed and Food Analysis laboratory within 24 h.

#### Intestinal contents

Intestinal contents (*n* = 62) were collected from the slaughtered animals and the samples were placed in sterile containers. The containers were immediately placed in cooler boxes containing frozen freezer packs.

#### Water

Water samples used in the abattoirs (*n* = 75) were collected according to instructions in the Water Research Commission No TT117/99 (2000). Briefly, the tap was opened and water was allowed to run for 3 min, followed by filling sterile bottles to about three quarters full. The bottles containing water were immediately placed in cooler boxes containing frozen freezer packs.

#### Faeces

Freshly voided faeces (*n* = 400) from cattle in the local communities were collected in sterile containers as a parallel study to determine the potential risk of *Salmonella* during slaughter and among inhabitants of Vhembe district.

### Microbiological analysis

#### *Salmonella* isolation and identification

For carcass and hide sponges, the samples were mixed thoroughly with maximum recovery diluents. The fluid mixture from the hide and carcass sponges (25 mL) was inoculated into 225 mL of non-selective pre-enrichment liquid medium containing buffered peptone water (BPW) supplemented with 1% Tween 80 (1:10 v/v). For cattle faeces and intestinal contents, 10-g samples were inoculated into 90 mL of BPW supplemented with 1% Tween 80. The inoculated BPW was incubated aerobically at 37 ºC for 18 ± 2 h. After incubation, 1-mL aliquots of the samples from BPW were inoculated into Rappaport–Vassiliadis soy broth (RVS) and Tetrathionate broth (MKTT) (RV; Merck, Darmstadt, Germany), followed by incubation at 42 ºC for 24 h. Loopfuls of the broth were plated onto Xylose Lysine Desoxycholate agar (XLD), RapidSal, and Brilliant Green agar (BGA), followed by incubation at 37 ºC for 24 h. The plates were examined for the presence of typical *Salmonella* colonies and further identified on the basis of biochemical tests. Isolates that were positive for lysine decarboxylase, were motile, did not produce urease, produced hydrogen sulfide on triple sugar iron agar, fermented dulcitol, and had variable reactions to mannitol were identified as *Salmonella* spp.

Isolation of *Salmonella* from water samples was adopted from the Standing Committee of Analysts (2002; Hammarstrom & Ljutov 1954). Briefly, the bacteria in water were concentrated by passage through membrane filters. The membrane filters were transferred into BPW, followed by incubation at 37 ºC for 18 ± 2 h. A portion of broth (10 mL) was transferred into enrichment selective media (90 mL), followed by subculture onto XLD, RapidSal, and BGA. The plates were incubated at 37 ºC for 24 h and examined for colonies that are typical of *Salmonella* species.

For all samples, internal quality control was performed in parallel with the test samples and involved the use of *Salmonella* Typhimurium ATCC14028 as positive control and *Escherichia coli* ATCC 25922 as a negative control. In addition, for water samples, blank control filters containing sterile distilled water (100 mL) and Ringer’s lactate solution were included. This was done after normal sterilisation of the filtration unit.

Colonies that were pink and black centred on XLD, pink on BGA, and purple on RapidSal agar (Merck, Darmstadt, Germany) were considered typical of *Salmonella* spp and were subcultured onto blood tryptose agar (BTA), followed by incubation at 37 ºC for 24 h. The pure cultures were confirmed using the following biochemical tests: Triple sugar iron agar, urea agar, malonate broth, phenol red dulcitol broth, lysine decarboxylase broth, decarboxylase broth control, and thio-gelatine semisolid agar to test for motility. *Escherichia coli* ATCC 25922 and *Salmonella* Typhimurium ATCC 14028 were included as negative and positive controls respectively. Isolates that were confirmed as *Salmonella* were preserved in nutrient broth supplemented with 35% glycerol and stored at -20 °C for subsequent species-specific polymerase chain reaction (PCR), antimicrobial susceptibility testing, and serotyping.

#### *Salmonella* Serotyping

*Salmonella* serotyping was done as prescribed in the Kauffman–White scheme. Briefly, the *Salmonella* isolates were tested against polyvalent and monovalent antisera for the presence of agglutination. The bacteria were tested for the presence of somatic (O) antigens, flagellar (H), and Vi antigens.

#### Antimicrobial susceptibility test

*Salmonella* isolates (*n* = 92) were tested for antimicrobial susceptibility towards the following antimicrobial agents: ampicillin (AMP), cefotaxime (CXT), enrofloxacin (ENR), kanamycin (K), and oxytetracycline (OT) using the Kirby–Bauer disk diffusion method. Briefly, the *Salmonella* spp were suspended in physiological saline until the turbidity was equivalent to 0.5 McFarland standard. The bacterial suspensions were inoculated onto Mueller Hinton agar, and disks were placed on the inoculated agar. The inoculated plates were incubated at 37 ºC for 24 h. The inhibition zones were measured using calipers and results were interpreted as sensitive, intermediate, or resistant according to Clinical Laboratory Standards. The reference strains of *E. coli* ATCC 25922 and *Salmonella* Typhimurium ATCC 14028 were included alongside the field isolates.

### Molecular identification

The PCR was used to confirm identification made by phenotypic tests.

#### DNA extraction

The *Salmonella* isolates were resuscitated by inoculation into nutrient broth, followed by incubation at 37 ºC for 2 h. Loopfuls from the nutrient broth were streaked onto nutrient agar, followed by incubation at 37 ºC for 24 h. The DNA was extracted from *Salmonella* colonies using the cell lysis method. Briefly, *Salmonella* cells were suspended in 1 mL of sterile distilled water. The bacterial suspensions were boiled at 99 ºC for 10 min, followed by cooling at room temperature and centrifugation at 13 000 rpm for 5 min. The supernatants were transferred into clean sterile eppendorf tubes and debris was discarded. The crude supernatant was used as DNA template in the PCR reactions.

#### Polymerase chain reaction

The 25-µL PCR reactions contained 12.5 µL DreamTaq master mix (Fermenats; Ontario, Canada), 10 µM of each primer targeting the *invA* gene (invAF-5’-GTGAAATTATCGCCAC GTTCGGGCAA-3’; InvAR–5’-TCATCGCACCGTCAAAGG AACC-3’ (Marlony *et al*. 2003), crude DNA extract (5 µL), and molecular grade water (5.5 µL). Part of the *invA* gene has been shown to be specific for *Salmonella*, and if detected, it may be used to confirm the genus (Nucera *et al*. 2006; Rahn *et al*. 1992). The PCR mixture was amplified in a thermocycler (Eppendorf, Hamburg, Germany) using the following conditions: denaturation at 95 ºC for 5 min, 35 cycles of denaturation at 95 ºC for 1 min, annealing at 56 ºC for 30 sec, and elongation at 72 ºC for 1 min. Final extension was done at 72 ºC for 7 min. *E. coli* ATCC 25922 and *Salmonella* Typhimurium ATCC 14028 were included as negative and positive controls, respectively. In the negative PCR control, molecular grade water was used instead of DNA.

#### Agarose gel electrophoresis

The PCR amplicons were subjected to electrophoresis through 1.5% ethidium bromide stained agarose gel at 3 V/cm for 1 h. A 100 bp molecular weight marker was used for determining the size of amplicons. Gels were visualised under ultraviolet light and the results were recorded using a gel documentation system (BIO-RAD; Hercules, CA, USA).

## Ethical consideration

This study was approved by the Agricultural Research Council. Dr Loock and Dr Mampane of Limpopo Provincial Veterinary Services offered permission to collect samples from rural abattoirs with the assistance of Mr Muthapuli.

## Results

### *Salmonella* species isolated

Table 1 shows a summary of the frequency of isolation of *Salmonella* on hides, carcasses, and intestinal contents and the associated serovars. On average, the frequency of *Salmonella* isolation on hides, carcasses, and intestinal contents was 35.37% (*n* = 81). Most of the *Salmonella* were isolated from hides (59.70%; 40/67), followed by carcasses (30%; *n* = 30). The frequency of occurrence of *Salmonella* in intestinal contents was 17.74% (11/62). All the *Salmonella* isolates had the *invA* gene successfully amplified.

**TABLE 1:** Distribution of *Salmonella* serovars according to samples types.

Samples	Hides†		Carcasses‡		Intestinal Contents§		Faeces¶	
			
	*n*	%	*n*	%	*n*	%	*n*	%
SAb	0	0.0	0	0	1	1.6	0	0.00
SA	1	1.5	0	0	0	0.0	0	0.00
SC	0	0.0	1	1	0	0.0	0	0.00
SE	0	0.0	1	1	4	6.5	3	0.75
SHa	1	1.5	0	0	1	1.6	0	0.00
SHe	1	1.5	0	0	1	1.6	0	0.00
SM	1	1.5	1	1	0	0.0	0	0.00
SN	1	1.5	1	1	0	0.0	0	0.00
SO	0	0.0	0	0	1	1.6	0	0.00
SP	1	1.5	0	0	0	0.0	0	0.00
ST	1	1.5	0	0	0	0.0	0	0.00
SS	0	0.0	1	1	0	0.0	0	0.00
Other	33	49.2	25	25	3	4.8	8	2.00
**Total Pos (%)**	**40**	**59.7**	**30**	**30**	**11**	**17.7**	**11**	**2.75**

Sab, *Salmonella* Aberdeen; SA, *Salmonella* Anatum; SC, *Salmonella* Cardoner; SE, *Salmonella* Enteritidis; SHa, *Salmonella* Hayindongo; SHe, *Salmonella* Heidelberg; SM, *Salmonella* Mbandaka; SN, *Salmonella* Nigeria; SO, *Salmonella* Othmarschen; SP, *Salmonella* Pretoria; SS, *Salmonella* Softenberg; ST, *Salmonella* Tennesse; Others, *Salmonella* spp.

† *n* = 67;

‡ *n* = 100;

§ *n* = 62;

¶ 400.

No *Salmonella* was isolated from the 75 water samples. Out of the 400 freshly voided cattle faeces that were tested, 2.75% (*n* = 11) were positive for *Salmonella*.

### *Salmonella* serovars

Overall, out of the 92 *Salmonella* spp isolated from hides, carcasses, intestinal contents, and freshly voided faeces, the predominant serovar was *S.* Enteritidis (*n* = 8; 8.7%). Other serovars that were identified include, *S.* Heidelberg (*n* = 2; 2.2%), *S.* Aberdeen (*n* = 1; 1.1%), *S.* Hayindongo (*n* = 1; 1.1%), *S*. Mbandaka (*n* = 2; 2.2%), *S.* Anatum (*n* = 2; 2.2%), *S*. Othmarschen (*n* = 1; 1.1%), *S.* Nigeria (*n* = 2; 2.2%), *S.* Tennessee (*n* = 1; 1.1%), *S*. Cardoner (*n* = 1; 1.1%), *S*. Senftenberg (*n* = 2; 2.2%), and *S*. Pretoria (*n* = 1; 2.2%). The remainder of the isolates could not be serotyped to serovar level. These *Salmonella* isolates belonged to OMD and OME serogroups because they reacted to these anti-*Salmonella* polyvalent somatic antisera. There were no monovalent antisera to further confirm the serovars of *Salmonella* isolates that belonged to these groups. The distribution of *Salmonella* serovars is summarised in Table 1.

### Antimicrobial resistance patterns

Overall, 66 (71.7%) of the *Salmonella* isolates were resistant to at least one of the tested antimicrobial agents resulting in 14 resistance patterns (Table 2). The serovars that were associated with the various resistance patterns are included in Table 2. Most *Salmonella* were resistant to oxytetracycline (51.90%), followed by ampicillin (39.24%), kanamycin (29.11%), cefotaxime (26.58%), and enrofloxacin (11.39%). Multidrug resistance (resistance to ≥ 3 antimicrobials) was observed in 25.32% of the *Salmonella* isolates.

**TABLE 2:** Summary of antimicrobial resistance patterns of *Salmonella* isolates from this study.

Antimicrobial resistance pattern	*Salmonella* Serovars	Number of isolates
AMP/CTX/K/OT/ENR	*S.* Cardoner	1
	*Salmonella* spp.	1
AMP/CTX/K/OT	*S.* Mbandaka	1
	*S.* Aberdeen	1
	*Salmonella* spp.	14
CTX/K/OT/ENR	*S.* Anatum	1
	*Salmonella* spp.	1
AMP/OT/ENR	*Salmonella* spp.	3
AMP/ ENR	*Salmonella* spp.	2
K	*Salmonella* spp.	2
AMP	*Salmonella* spp.	9
ENR	*Salmonella* spp.	0
AMP/OT	*Salmonella* spp.	1
OT	*S.* Enteritidis	8
	S. Heidelberg	1
	*Salmonella* spp.	11
CTX/OT	*Salmonella* spp.	2
AMP/K	*Salmonella* spp.	3
CTX	*Salmonella* spp.	2
K/OT	*S.* Anatum	1
OT/ENR	*Salmonella* spp.	1

The *Salmonella* spp. could not be serotyped to serovar level using the available panel of antisera. They were identified as belonging to groups OMD and OME.

AMP, ampicillin; CTX, cefotaxime; K, kanamycin; OT, oxytetracycline; ENR, enrofloxacin; *S., Salmonella*.

## Discussion

The aim of this study was to determine the contamination of hides, carcasses, and intestinal contents of cattle slaughtered in 23 rural abattoirs of Vhembe district, South Africa by *Salmonella* serovars, with a view to determining potential sources of contamination during cattle slaughter. All the sample types, with the exception of water were contaminated with *Salmonella* to varying degrees. The serovars were diverse, and most of the *Salmonella* belonged to group OMD and OME and they were not typed to serovar level. Numerous *Salmonella* serovars belong to OMD and OME groups; hence it is challenging to establish the public health significance of these isolates. Some *Salmonella* serovars that were observed on the cattle carcasses were not necessarily observed on the hides or intestinal contents, suggesting other potential contamination sources that were not analysed in this study. Despite extensive cattle production in rural areas, most of the *Salmonella* isolates were resistant to at least one of the tested antimicrobials and multidrug resistance was also observed.

The hides, carcasses, and intestinal contents of cattle in this study showed notable levels of *Salmonella* contamination. *Salmonella* isolates on the hides are usually transferred from the environment or from faecal contamination during transportation of the animals to the abattoir (Antic *et al*. 2010). The contamination of hides by *Salmonella* that was observed in this study is not unusual because it is often not practical to control *Salmonella* at the farm level; therefor, contaminated cattle are usually presented for slaughter (Antic *et al*. 2010; Bacon *et al*. 2000; Small *et al*. 2002; Vivas & Buncic 2004). Studies involving control measures for decontamination of hides have been done, but the practicalities are uncertain (Mies *et al*. 2004). However, it is plausible to present relatively clean animals for slaughter to minimise the risk of carcass contamination during slaughter.

The presence of *Salmonella* in intestinal contents could be related to the asymptomatic carrier status of some cattle that continue to shed *Salmonella* without showing any clinical signs (Cummings *et al*. 2010). This could result in presentation of contaminated animals for slaughter, which poses a risk of transfer on carcasses. Information on the prevalence of *Salmonella* carrier status in African cattle is limited. In Ethiopia, *Salmonella* carrier status was observed to be 7.07% and 43.81% among cattle and pigs respectively (Tadesse & Tessema 2014). These asymptomatic animals may become a source of spreading *Salmonella* in the herd and transmission of foodborne salmonellosis in humans.

The proportion of carcass contamination in this study was relatively high and the potential sources of contamination were diverse. The carcasses could have been contaminated during hide removal or during evisceration. Although molecular epidemiology tools such as pulsed-field gel electrophoresis and whole genome sequence analysis were not used to prove the unequivocal similarity of strains, it is highly likely that *Salmonella* Mbandaka and *Salmonella* Nigeria that were isolated from carcasses could be a result of cross-contamination from hides. Likewise, the *S.* Enteritidis on carcasses could have been transmitted from intestinal contents and faecal matter. However, *S.* Enteritidis is not regularly isolated in cattle. Nevertheless, 6 out of 232 *S.* Enteritidis from cattle, poultry, sheep, goats, and pigs were isolated from cattle in a study that was undertaken in South Africa from 1999 to 2006 (Kidanemariam *et al*. 2010). Use of the same equipment for slaughtering different animals and inadequate sterilisation of the utensils, which was observed during sampling, could have contributed to high chances of *Salmonella* contamination. In some of the abattoirs, workers sharpened knives that were used during slaughter on unconventional objects such as stones despite the potential risk of contamination. It would be paramount to study the role of such practises in *Salmonella* contamination of cattle carcasses. This was outside the scope of this study and constitutes a limitation of the study. Some unconventional slaughter practises in a small proportion of the rural abattoirs could have also exacerbated the frequency of carcass contamination. For instance, despite the provisions of the procedure for cattle slaughter that are elaborated in the *Meat Safety Act* (No. 40 of 2000), some workers in rural abattoirs still used the head of the animal as floor-support during hide removal, which increases the chances of contamination. Such unhygienic practises lead to cross-contamination by foodborne pathogens including *Salmonella*. This could have contributed to the inconsistent diversity of *Salmonella* serovars that were observed on hides, carcasses, and intestinal contents.

The separation of ‘clean’ and ‘dirty’ areas is usually proposed as one of the control measures to curb cross-contamination. However, separating ‘clean’ and ‘dirty’ areas may be extremely challenging in rural abattoirs from this study because animal slaughter took place in one room without compartments. This set-up highlights the need for strict physical and chemical decontamination programmes and regular inspection of the slaughter process as well as monitoring of the effectiveness of refrigeration of final carcass to minimise proliferation of *Salmonella* (Sofos & Geornaras 2010). The use of chemical decontamination is not always acceptable in different geographical areas, and in some cases, the use of chemicals needs to be followed by rinsing of the chemicals with water (Hugas & Tsigarida 2008). Little attention has been focused on the use of biological control such as competitive microbial cultures and bacteriophages. Despite the potential effectiveness of physical, chemical, or biological control measures, the starting-point for producing safe food should be based on good hygienic practice and good manufacturing practice that are underpinned by hazard analysis critical control points management principles (Nørrung & Buncic 2008). In addition, training of both food handlers and consumers plays an important role in overall food safety.

Our findings are in contrast with a recent study that was conducted in abattoirs that slaughter cattle and pigs in Vhembe district, South Africa where *Salmonella* were not isolated (Tanih *et al*. 2015). The different findings could be because of the variation in number of abattoirs, source of animals, and types of samples. In addition, different isolation approaches were used. Compared to Tanih *et al*. (2015), our protocol used a relatively larger sample volume (25 mL) for pre-enrichment in buffered peptone water. In addition, this study used both MKTT and RVS for selective enrichment and XLD, BGA, and RapidSal agar for culture that increases the likelihood for the recovery and detection of *Salmonella* species.

In the current study, it was demonstrated that *Salmonella* isolates were shed in faeces of cattle from communities in areas that supply rural abattoirs in Vhembe district, which poses a potential environmental health risk. This is particularly important because it was observed that cattle manure was used for boosting growth of vegetables such as cabbage, which may be consumed raw as salads. In addition, *Salmonella-*infected cattle may pose a risk as they may cause cross-contamination during slaughter.

In this study, diverse *Salmonella* serovars were isolated from cattle hides, carcasses, intestinal contents, and faeces. *S.* Anatum, *S.* Tennessee, and *S.* Pretoria were isolated from hides, but were not isolated from the carcass and intestinal contents. This indicates possible environmental contamination. In a similar study, *S.* Anatum was isolated from hides, faeces, and subiliac lymph nodes (Gragg *et al*. 2013). *Salmonella* Anatum was the predominant serovar found in a study in Ethiopia that was aimed at determining antimicrobial resistance profiles and *Salmonella* serovars in slaughterhouse personnel, the environment in the slaughterhouse, and beef cattle (Sibhat *et al*. 2011). Although *S.* Anatum, *S.* Tennessee, and *S.* Pretoria have been rarely linked to clinical cases, *S*. Anatum was implicated in the infection of a patient who consumed contaminated unpasteurised orange juice (Krause, Terzagian & Hammond 2001). *Salmonella* Tennessee was deemed the causative agent of a nationwide outbreak of salmonellosis in the USA, which was likely linked to environmental contamination of a peanut butter plant (Sheth *et al*. 2011). *Salmonella* Enteritidis was found on one of the carcasses, four intestinal contents, and three cattle faecal samples. It is likely that the *S.* Enteritidis was transmitted from the intestinal contents, but this finding needs to be confirmed by strain typing techniques such as pulsed-field gel electrophoresis and whole genome sequence analysis. Globally, salmonellosis in humans has been associated with *S.* Enteritidis and *S*. Typhimurium (Hendriksen *et al*. 2011). The presence of *S*. Cardoner and *S*. Senftenberg on two carcasses from this study, but not on the hides or intestinal contents, highlights the presence of other sources of contamination that were not analysed in this study. Although the information about clinical cases related to *S*. Cardoner and *S*. Senftenberg is scant, it is paramount to be vigilant about hygiene in order to reduce the risk of infection, particularly among individuals who may be immunocompromised.

Most *Salmonella* in this study were resistant to OT. Tetracycline resistance among food production animals has been attributed to selection pressure exerted from diverse sources such as prophylaxis, veterinary therapy, and use of antibiotics for animal growth promotion (Chopra & Roberts 2004; Khachatourian 1998). The mechanisms of antimicrobial resistance may be broadly divided into genetic and phenotypic. Genetic resistance may be because of chromosomal mutation, or acquired genes that are harboured on transposons or plasmids (Khachatourian 1998). Tetracycline resistance may occur through tetracycline modification, ribosome protection, and tetracycline efflux (Chopra & Roberts 2004).

Multidrug resistant *Salmonella* were predominant in this study. This finding is significant because antimicrobial resistance carrying plasmids could be co-localised with virulence genes, which enhances invasiveness of the bacteria (Brichta-Harhay *et al*. 2011; Helms, Simonsen & Molbak 2004). This could further complicate management of infection associated with multidrug resistant *Salmonella*. Multidrug resistant foodborne pathogens from carcasses highlight the risk associated with the challenge of treating both human and animal infections. The situation may be exacerbated in immunocompromised individuals who are usually highly susceptible. Our findings are in harmony with observations of multidrug resistant *Salmonella* isolated from meat and environmental sources in other studies (Poppe *et al*. 2005).

The presence of multidrug resistant *Salmonella* pathogens warrants further investigation into general cattle farming practices and handling of veterinary drugs that might contribute to selection pressure in animals that are raised using extensive farming. The results further warrant a holistic and multidisciplinary approach to biosecurity and safety. It would be interesting to establish if there is any association between the antimicrobial resistance and virulence genes in the *Salmonella* isolates from this study in future. It is important to determine the serovars of all OMD, OME *Salmonella* groups for epidemiological purposes. In addition, it is paramount to establish the *Salmonella* pulsotypes and compare the patterns to those found in humans and other geographical areas. Further studies are required to study the contamination of cattle carcasses by *Salmonella* and the different serovars that are involved in different regions of South Africa. It would be important to determine the link between similar serovars using technologies with higher resolution such as whole genome sequence analysis.

## Conclusion

The hides of cattle presented for slaughter in rural abattoirs of Vhembe district are highly contaminated with *Salmonella* and this may pose a risk of carcass contamination during slaughter. Some asymptomatic cattle presented for slaughter contribute to carcass contamination because of *Salmonella* in the intestines that has a high chance of being transferred onto the carcass. This could be exacerbated by not following proper slaughter procedure in some of the abattoirs. The differences among *Salmonella* serovars on hides, carcasses, and intestinal contents illustrates that there are other sources of contamination during slaughter. Antimicrobial resistance among *Salmonella* from cattle and environmental samples of Vhembe district was high. This poses a risk to consumers because the *Salmonella* may proliferate along the food value chain. Together, there is a need for regular assessment and inspection during animal slaughter in Vhembe district rural abattoirs.
